# Overexpression of the DEC1 Protein Induces Senescence In Vitro and Is Related to Better Survival in Esophageal Squamous Cell Carcinoma

**DOI:** 10.1371/journal.pone.0041862

**Published:** 2012-07-23

**Authors:** Qing Xu, Peiqing Ma, Chenfei Hu, Lechuang Chen, Liyan Xue, Zaozao Wang, Mei Liu, Hongxia Zhu, Ningzhi Xu, Ning Lu

**Affiliations:** 1 Laboratory of Cell and Molecular Biology and State Key Laboratory of Molecular Oncology, Cancer Institute and Cancer Hospital, Chinese Academy of Medical Sciences and Peking Union Medical College, Beijing, China; 2 Department of Pathology, Cancer Institute and Cancer Hospital, Chinese Academy of Medical Sciences and Peking Union Medical College, Beijing, China; National Cancer Institute, National Institutes of Health, United States of America

## Abstract

Esophageal squamous cell carcinoma (ESCC) is a leading cause of cancer-related death in China and has limited effective therapeutic options except for early surgery, since the underlying molecular mechanism driving its precursor lesions towards invasive ESCC is not fully understood. Cellular senescence is the state of the permanent growth arrest of a cell, and is considered as the initial barrier of tumor development. Human differentiated embryo chondrocyte expressed gene 1 (Dec1) is an important transcription factor that related to senescence. In this study, DEC1 immunohistochemical analysis was performed on tissue microarray blocks constructed from ESCC combined with adjacent precursor tissues of 241 patients. Compared with normal epithelia, DEC1 expression was significantly increased in intraepithelial neoplasia and DEC1 expression was significantly decreased in ESCC in comparison with intraepithelial neoplasia. *In vitro*, DEC1 overexpression induced cellular senescence, and it inhibited cell growth and colony formation in ESCC cell line EC9706. Fresh esophagectomy tissue sections from five ESCC patients were detected by immunohistochemistry of DEC1 and senescence-associated β-galactosidase (SA-β-Gal) activity, and strongly positive expression of DEC1 was correlated to more senescent cells in these fresh tissue sections. Kaplan – Meier method analysis of the 241 patients revealed that DEC1 expression levels were significantly correlated with the survival of ESCC patients after surgery. The expression levels of DEC1 were also correlated with age, tumor embolus, depth of invasion of ESCC, lymph metastasis status and pTNMs. These results suggest that DEC1 overexpression in precursor lesions of ESCC is a protective mechanism by inducing cellular senescence in ESCC initiation, and DEC1 may be a potential prognostic marker of ESCC.

## Introduction

Esophageal cancer is one of the most common malignancies in the world. It was estimated that 482,300 new esophageal cancer cases and 407,000 deaths occurred in 2008 worldwide, which ranked eighth in cancer incidence and six in cancer mortality [1]. There are two major types of esophageal cancer, esophageal adenocarcinoma (EAC) and esophageal squamous cell carcinoma (ESCC). Although incidence rates for EAC have been increasing in some western countries, ESCC still predominates over EAC, especially in the “esophageal cancer belt”, where stretching from the northern Iran to the North-Central China, and accounting for 90% of esophageal cancer cases [2]. Histologically, the evolution of ESCC involves a multistage process, in which normal esophageal squamous epithelia undergo both genetic and histological changes into noninvasive precursor lesions, including low grade intraepithelial neoplasia and high grade intraepithelial neoplasia, and then towards invasive carcinoma [3], [4]. It was reported that different relative risks of precursor lesions of ESCC, suggesting that some certain events occur during this process [5], [6]. Despite of extensive research on this process, which molecules or biological events regulate the initiation and progression of ESCC remains obscured [4], [7], [8].

Cellular senescence is defined as the state of stable growth arrest of a cell, which was proposed in 1960s by Leonard Hayflick, who observed that normal fibroblasts would cease to proliferate and still viable for several weeks [9], [10]. It is now clear that the reason for replicative senescence is telomere attrition [11]. Besides, activation an oncogene or inactivation of certain tumor suppressor gene, DNA damage, oxidative stress, some chemotherapeutic drugs, radiation and cell reprogramming could also induce cell senescence *in vitro* [12], [13], [14]. Because of the abnormal stimulation, all these kinds of senescence are called premature senescence or accelerated senescence to distinguish from replicative senescence. Until recently, evidence that cellular senescence happens *in vivo* emerged and the role of senescence in human body has been deeply understood [15], [16]. Senescence markers, such as senescence-associated β-galactosidase (SA-β-Gal), senescence-associated heterochromatin foci (SAHF), DEC1, DCR2, and p15^ INK4b^, were explored both in culture and *in vivo*, however, none of them are exclusive to senescent cells [17]. Several studies have confirmed that senescence is the initial barrier in cancer development because senescent cells have been found in precancerous lesions and premalignant tumors [15], [18], [19], [20], [21]. Meanwhile, the role of senescence in ESCC development still remains to be investigated.

Human differentiated embryo chondrocyte expressed gene 1 (Dec1), also known as BHLHE40/Bhlhb2/Stra13/Sharp-2, is a basic helix-loop-helix transcription factor that involves in circadian rhythms, cell proliferation, differentiation, apoptosis, and cellular senescence [22], [23], [24], [25]. It is wildly used as a marker of senescence *in vivo*, and it could directly induce senescence in cultured cells [25], [26], [27]. DEC1 is wildly expressed in most normal human tissues, but only in a proportion of cells, while in tumors, expression of DEC1 is thought to be induced by hypoxia and was detected up-regulated in several kinds of malignancies, including breast, stomach, colon, lung, liver, and pancreatic ductal adenocarcinoma [28], [29], [30], [31], [32], [33], [34]. However, DEC1 expression in ESCC has not been investigated yet, and could DEC1 play the same role in ESCC as in above-mentioned cancers?

Based on what we have done on ESCC before and what we have learnt about DEC1, we try to test the hypothesis, that DEC1 differently expressed in normal esophageal epithelia, ESCC and its precursor lesions, and the cellular senescence might play an important role in preventing the development of ESCC, in the present study.

## Results

### DEC1 expression in normal esophageal squamous epithelia, intraepithelial neoplasia, and ESCC detected by immunohistochemistry

**Figure 1:**
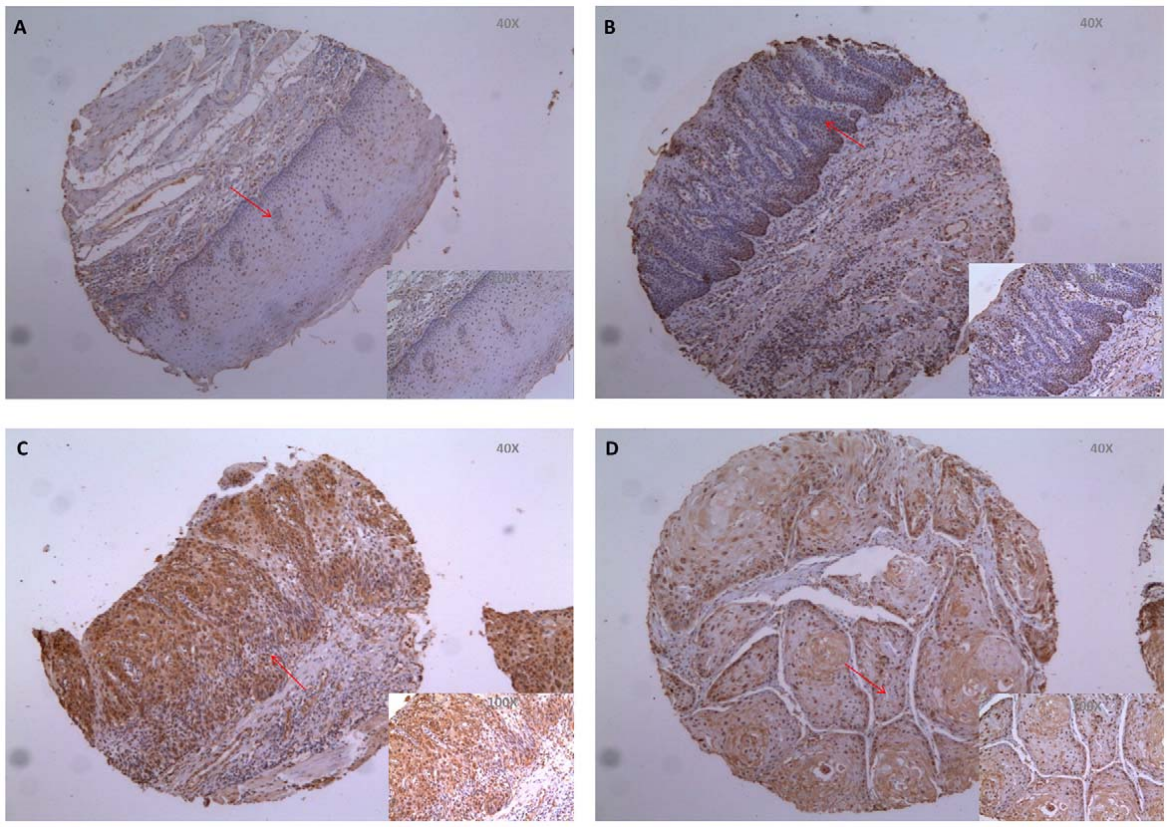
Representative photographs of DEC1 expression in different lesions by immunohistochemistry. Red arrows indicate the enlarged parts. (A) Normal esophageal epithelia. (B) Low grade intraepithelial neoplasia. (C) High grade intraepithelial neoplasia. (D) ESCC.

DEC1 expression in normal esophageal squamous epithelia and different lesions of ESCC development was summarized in Table 1. Compared with normal epithelia, DEC1 expression was significantly increased in low grade intraepithelial neoplasia (p<0.001), and there was also a significant difference in DEC1 expression between low grade intraepithelial neoplasia and high grade intraepithelial neoplasia (p = 0.047). However, DEC1 expression was significantly decreased in ESCC in comparison with high grade intraepithelial neoplasia (p<0.001). In normal esophageal squamous epithelia, DEC1 was predominately negative expression (63.0%) (Fig. 1A). Then both the rate and intensity were increased along with the severities of consecutive precursor lesions (Fig. 1B and C). At last, there was an obvious decrease of DEC1 expression in ESCC (Fig. 1D).

**Table 1:** Summary of immunohistochemical expression of DEC1 in different lesions.

Protein		Normal esophageal epithelia	Low grade intraepithelial neoplasia	High grade intraepithelial neoplasia	ESCC	Value		
						*p* a	*p* ^b^	*p* ^c^
**DEC1**	**−**	131 (63.0%)	35 (24.0%)	20 (13.7%)	80 (39.8%)			
	**+**	72 (34.6%)	56 (38.4%)	55 (37.7%)	73 (36.3%)			
	**++**	5 (2.4%)	55 (37.7%)	71 (48.6%)	48 (23.9%)	0.000	0.047	0.000

a Esophageal normal epithelia VS. Low grade intraepithelial neoplasia. ^b^ Low grade intraepithelial neoplasia VS. High grade intraepithelial neoplasia. ^c^ Low grade intraepithelial neoplasia VS. ESCC.

### Overexpression of DEC1 induces cellular senescence and inhibits cell growth in vitro

**Figure 2:**
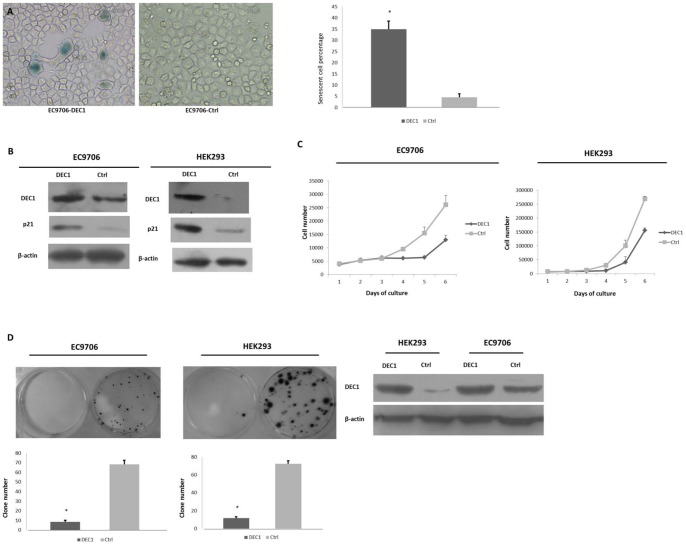
DEC1 induces cellular senescence and inhibits cell growth in vitro. (A) SA-β-Gal staining assay was performed and senescent cell percentage was counted 48 h after transfection in EC9706 cells. (B) Protein expressions were analyzed by western blotting 48 h after transfection in EC9706 and HEK293 cells. (C) Growth curves of EC9706 and HEK293 cells after transfection with pCMV6-XL5-DEC1 and control plasmids. (D) Cells were seeded 24 h after transfection and colony number was counted two weeks G418 selection. Cotransfection efficiency was analyzed by western blotting. (*, P<0.05 as compared with ctrl.).

It has been shown that DEC1 could induce cellular senescence in both normal and cancer cell lines and could cause proliferation inhibition in both normal and cancer cell lines [25], [26], [31]. We thus speculated that DEC1 induces premature senescence in ESCC cell line. To test this, we transfected DEC1 into EC9706 and confirmed that overexpression of DEC1 induces senescence by SA-β-Gal staining assay and the typically enlarged and flattened cell shape. (Fig. 2A) [35]. Western blot analysis showed that p21**^WAF1/CIP1^**, one of the most important proteins that mediates cellular senescence [27], was also increased by ectopic expression of DEC1 (Fig. 2B). For HEK293 cell line is relative normal epithelial and its growth could be inhibited by DEC1 overexpression [31], we used it as a positive control. Then we found DEC1 overexpression significantly suppressed the growth of EC9706 and HEK293, as measured by growth rate and colony formation assay (Fig. 2C and D).

### Overexpression of DEC1 is correlated to more senescent cells in fresh tissue sections

**Figure 3:**
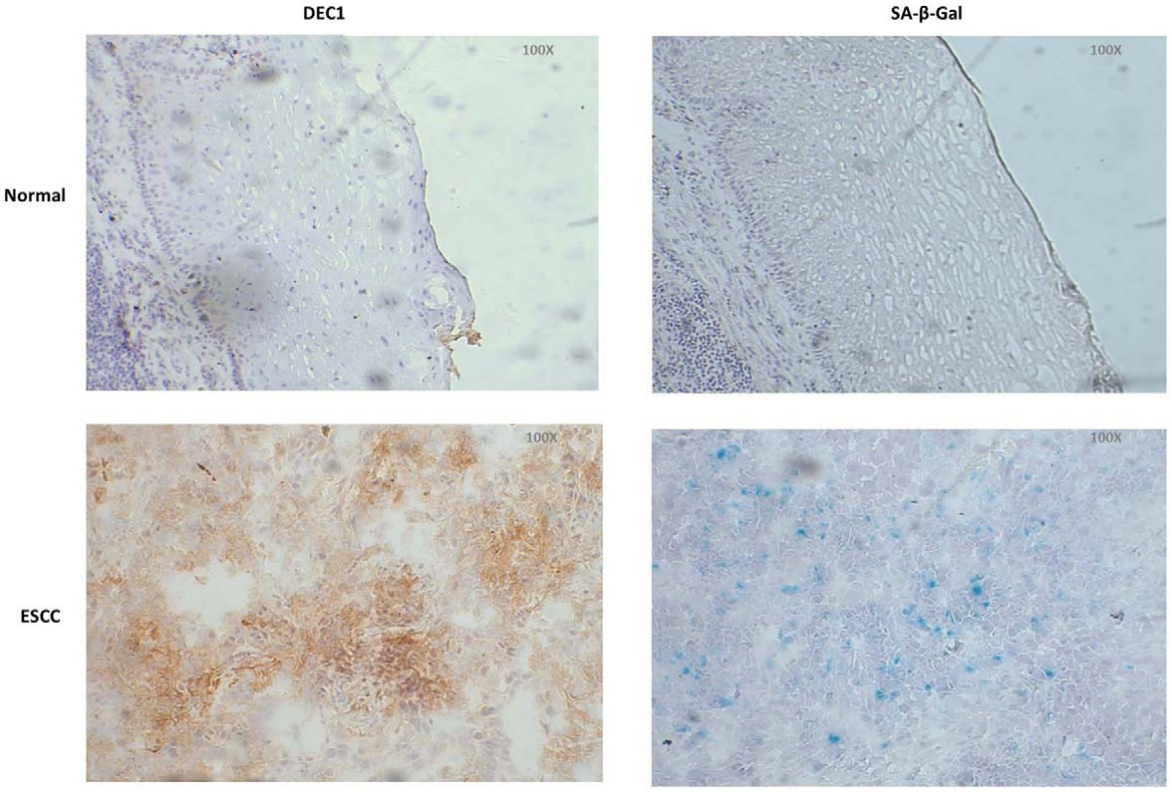
Detection of DEC1 and SA-β-Gal activity in fresh tissue sections. DEC1 expression and SA-β-Gal activity in ESCC and adjacent normal epithelia were detected by immunohistochemistry and SA-β-Gal staining assay in consecutive frozen sections.

To validate our observation that DEC1 was related to cellular senescence *in vitro*, we looked for fresh esophagectomy specimens of ESCC patients. By comparing the expression of DEC1 and SA-β-Gal staining in consecutive frozen sections, it was confirmed that strong expression of DEC1 was correlated to more senescent cells *in vivo* (Fig. 3).

### Correlation of DEC1 expression in ESCC with clinicopathological characteristics and survival

**Figure 4:**
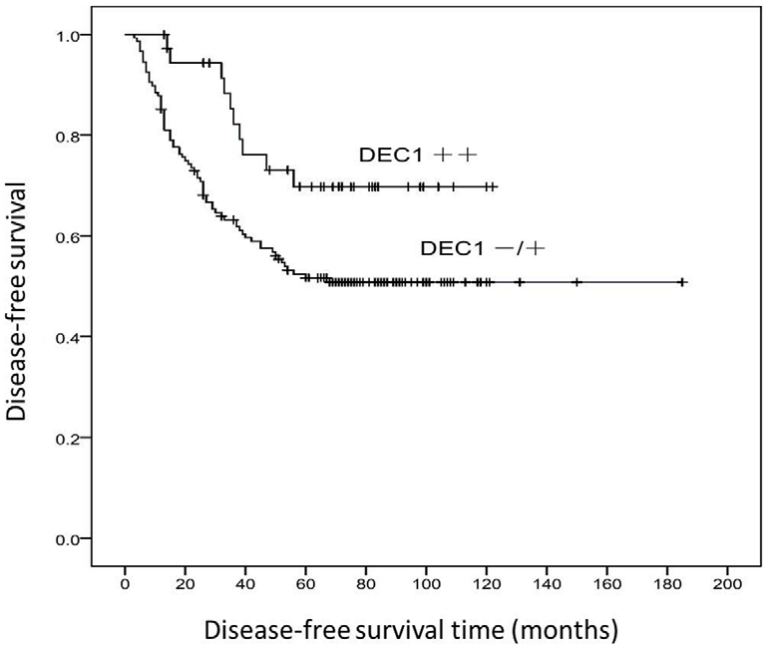
Survival curves of DEC1 expression in ESCC patients analyzed by Kaplan-Meier method.

Table 2 shows the association between DEC1 expression with clinicopathological characteristics in ESCC. There was a significant correlation between DEC1 expression and the tumor embolus (p<0.001), depth of invasion of ESCC (p<0.001), lymph metastasis status (p<0.001) and pathological Tumor-Node-Metastasis (p<0.001). The expression of DEC1 were also correlated with age (p = 0.025), with higher expression in the patients <60 years old than those ≥60 years old. However, no significant associations were observed with patients' gender (p = 0.787), parts of occurrence (p = 0.436), and tumor differentiation (p = 0.614). We also analyzed the relation between DEC1 expression and survival. Kaplan-Meier method analysis revealed that DEC1 expression levels were significantly correlated with the survival of ESCC patients after surgery (p = 0.025), with the five-year survival rate is 51.7% for patients of DEC1 negative or weakly positive expression versus 69.7% for patients of DEC1 strongly positive expression (Fig. 4).

**Table 2:** Summary of correlation of DEC1 expression with clinicopathological characteristics in ESCC.

	DEC1					
	-		+	++	χ^2^	*p* value
**Age**						
** <60**	34 (31.8%)		47 (43.9%)	26 (24.3%)		
** ≥60**	46 (48.9%)		26 (27.7%)	22 (23.4%)	7.364	0.025
**Gender**						
** Male**	59 (40.4%)		54 (37.0%)	33 (22.6%)		
** Female**	21 (38.2%)		19 (34.5%)	15 (27.3%)	0.480	0.787
**Part of thoracic portion**						
** Upper**	12 (40.0%)		9 (30.0%)	9 (30.0%)		
** Middle**	51 (40.5%)		43 (34.1%)	32 (25.4%)		
** Lower**	17 (37.8%)		21 (46.7%)	7 (15.6%)	3.782	0.436
**Differentiation**						
** Well**	15 (34.1%)		19 (43.2%)	10 (22.7%)		
** Moderate**	42 (44.7%)		29 (30.9%)	23 (24.5%)		
** Poor**	23 (36.5%)		25 (39.7%)	15 (23.8%)	2.673	0.614
**Tumor embolus**						
** Negative**	58 (34.7%)		61 (36.5%)	48 (28.7%)		
** Positive**	22 (64.7%)		12 (35.3%)	0 (0%)	16.162	0.000
**Depth of invasion**						
** T1**	13 (12.4%)		46 (43.8%)	46 (43.8%)		
** T2**	23 (67.6%)		10 (29.4%)	1 (2.9%)		
** T3**	44 (71.0%)		17 (27.4%)	1 (1.6%)	81.590	0.000
**Lymph metastasis**						
** Negative**	36 (30.3%)		42 (35.3%)	41 (34.5%)		
** Positive**	44 (53.7%)		31 (37.8%)	7 (8.5%)	20.422	0.000
**pTNMs** *						
** I**		7 (9.2%)	30 (39.5%)	39 (51.3%)		
** II a+ II b**		46 (53.5%)	31 (36.0%)	9 (10.5%)		
** III**		27 (69.2%)	12 (30.8%)	0 (0%)	70.693	0.000

* pTNMs, pathological Tumor-Node-Metastasis. (AJCC, 6^th^ edition).

## Discussion

Despite of intense study on etiology, epidemiology, pathogenesis of esophageal cancer, the development of esophageal cancer at molecular level remains unclear [36], [37]. In clinical pathology, the precursor lesions of ESCC are thought to consist of various morphological stages: mild dysplasia, moderate dysplasia, severe dysplasia and carcinoma *in situ*. The mild dysplasia and moderate dysplasia are also called low grade intraepithelial neoplasia, while severe dysplasia and carcinoma *in situ* are defined as high grade intraepithelial neoplasia [4], [38] (Fig. S1). The fact that the earlier detection of the precursor lesions of ESCC, the better survival makes people believe that early detection through potential biomarkers is an effective solution of ESCC. However, there was no such biological event could account for this multistage process while many molecules have been identified as preventive or prognostic biomarkers in precursor lesions [4], [5], [38], [39], [40]. We speculate that there should be some events that impede the initiation and development of ESCC, and thus one of the major purposes of this study is to address the issue.

Our knowledge of the relationship between cancer and senescence was greatly pushed forward as the discovery that overexpression certain oncogenes could induce premature senescence in vitro [41], [42], [43]. At present, cellular senescence is thought an important barrier to tumorigenesis *in vivo*, and bypassing senescence is crucial both for precancerous cells to become tumor cells [15], [44], and for cancer cells to evade therapeutic drugs and radiation [13], [45]. Though the role of senescence was studied in many kinds of cancer, senescence in ESCC and its precursor lesions remain to be investigated. Based on our data showed in this study, we supposed that senescence is the event that hinders the ESCC development. DEC1 is wildly used as a senescence biomarker *in vivo*, and could induce senescence itself in normal and cancer cell lines *in vitro* [25], [27], [46]. Thus it seems an ideal molecule to support our assumptions.

Here we demonstrate that DEC1 is differently expressed in ESCC and its precursor lesions, and overexpression of DEC1 induces senescence in EC9706 cell line, indicating that cellular senescence may play a protective role in ESCC initiation. The senescence signals are transduced mainly through p53–p21 and p16-pRB pathways [12], [17]. As a mediator of p53, we confirmed that DEC1 overexpression induces p21**^WAF1/CIP1^** expression in EC9706 cells. P21**^WAF1/CIP1^** expression in ESCC and its precursor lesions was also detected by our tissue microarray slides, with almost the same tendency of DEC1 (data no shown). Further, the correlation of DEC1 expression with clinicopathological characteristics and survival in ESCC makes DEC1 a promising diagnosis and prognostic factor for translational research.

However, several issues are still to be understood. Firstly, it is thought that precancerous cells must bypass senescence to become invasive cancer cells, and how this process happen *in vivo* remains unclear because it is difficult to get precancerous samples that definitely won't become cancer. Secondly, according to our results, whether overexpression of DEC1 caused senescence *in vivo* requires further investigate. Though DEC1 induces senescence *in vitro*, the evidence that DEC1 cause senescence *in vivo* is not enough, and it is more likely that senescence of esophageal cells make DEC1 overexpress, since so many of genes that can induce senescence change their expression in cancer and their precursor lesions, such as Ras, p15^INK4b^, and p16^INK4a^ [21], [47]. In addition, while further investigation is needed, it is possible that DEC1 expression was altered due to genomic instability, because Dec1 gene locate at 3p26, a hotspot of chromosome mutation in ESCC and other tumors [48], [49], [50]. At last, the role of cellular senescence in cancer is seen as a double-edged sword recently [51], [52]. That is while senescence halts cells to proliferation, senescent cells are potential malignant themselves and may trigger other cell growth [53], [54].

Taken all, although the detailed relationship between senescence and ESCC is to be confirmed further, our results show that DEC1 overexpression in precursor lesions of ESCC and DEC1 overexpression may serve as a protective mechanism by inducing senescence and a prognostic factor for good survival. Therefore, we envision that DEC1 may be an attractive target for therapy and prognosis of ESCC.

## Materials and Methods

### Tissue sample collection and tissue microarray construction

ESCC combined with adjacent precursor tissues from 241 patients who had receive esophagectomy were collected in Cancer Institute and Cancer Hospital, Chinese Academy of Medical Science and Peking Union Medical College from February, 1990 to January, 2004. There were 177 males and 64 females, and their age ranged from 37 to 81, with the mean of 57.6 years old. None of these patients had received radiotherapy and chemotherapy before surgery. Among the 241 patients, 79 cases were ESCC combined with low grade intraepithelial neoplasia, 79 cases were ESCC combined with high grade intraepithelial neoplasia, and 83 cases were ESCC combined with both precursor lesions. The clinicopathological characteristics of the patients were summarized in Table S1. There were 221 patients had complete follow-up data, and the follow-up period was from 3 months to 191 months, with a mean period of 59.1 months. The overall survival time was defined as the months from the date of surgery to the date of death or loss follow-up. For tissue microarray construction, representative areas by H&E staining which containing morphologically normal epithelia, defined precursor lesions and representative ESCC were circled on the glass and used as a template. The tissue microarray was constructed as previously described, using a manual Tissue Arrayer (Beecher Instruments, Silver Spring, MD) [55].

Frozen sections were obtained from fresh esophagectomy tissues of other five ESCC patients in September, 2011. The tissues were flash frozen in liquid nitrogen after resection, and then embedded in OCT compound and cut into 4 μm sections. Frozen sections were immediately used for immunohistochemistry and detection of SA-β-Gal activity.

### Ethics Statement

The study was approved by the medical ethics committee of Cancer Institute and Cancer Hospital, Chinese Academy of Medical Science and Peking Union Medical College. All tissue samples were collected as part of a diagnostic or therapeutic surgery after the patients gave written informed consent. Patients offering samples for the study were pseudonymized and analyzed anonymously, so no individual-related data are contained in the database.

### Immunohistochemistry and assessment

The standard streptavidin peroxidase (SP) method was performed for immunostaining. In brief, sections were cut at 4 μm from the tissue array blocks and dewaxed in xylene and rehydrated in serial dilutions of ethanol. Antigen retrieval was carried out in 0.01 M sodium citrate buffer (pH 6.0) for 10 minutes by microwave oven heating. Then endogenous peroxidase activity was blocked by 3% hydrogen peroxide for 10 minutes, and nonspecific staining was blocked by 10% normal goat serum (Vector Laboratories Inc.) for 10 minutes. The slides were incubated with anti-DEC1 (1∶100, Sigma) for 90 minutes, and then incubated with biotin-conjugated secondary antibody for 30 minutes, followed by incubation with streptavidin peroxidase for 15 minutes. 3, 3′- diaminobenzidine tetrachloride (DAB) was applied to identify peroxidase activity. Each incubation step was performed at room temperature and was followed by sequential washed for three minutes each for three times in PBS. Finally, sections were dehydrated in alcohol and cleared in xylene.

We scored DEC1 with the criteria that combined the rate of nuclear positive cells with intensity because the intensity of the protein was not uniform among different lesions [34]. The rate of positive cells was grade: 0, <5%; 1, 5∼25%; 2, 26∼50%; 3, 51∼75%; 4, >75%. Then, the intensity was graded as follows: 0, negative; 1, weak; 2, moderate; 3, strong. A final score was got by multiplication of the two scores above. Scores of 0∼4 were defined as “negative expression” (−); scores of 5∼8 as “weakly positive expression” (+), and scores of 9∼12 as “strongly positive expression” (++) [4].

### Cell lines, plasmids, and transfection

Human esophageal squamous cell line EC9706 was a generous gift from Prof. Mingrong Wang (Cancer Institute, Peking Union Medical College, Beijing, China) and was grown in RPMI 1640 medium (Gibco) [56]. HEK293 cell line was obtained from American Type Culture Collection, Manassas, USA, and was cultured in Dulbecco's modified Eagle's medium (Gibco). All medium contained 10% fetal bovine serum and supplemented with 100 U/mL penicillin and 100 μg/mL streptomycin. Cells were maintained at 37°C in a humidified incubator with 5% CO_2_.

The DEC1 expression plasmid pCMV6-XL5-DEC1 was purchased from OriGene Technologies and empty plasmid pcDNA3.1 (−)/myc-His B (Invitrogen) was used as control plasmid. Transfection was performed in 80% confluent cells using Lipofectamine 2000 Reagent (Invitrogen) according to the manufacturer's protocol.

### Western blotting

Transfected cells in 6- well plates were harvested and lysed in RIPA buffer (Sigma). Proteins were separated by SDS-PAGE and transferred to nitrocellulose membranes. Primary antibodies against DEC1 (Santa Cruz), p21 (Cell Signaling Technology), β-actin (Sigma) and horseradish peroxidase conjugated secondary antibodies (Zhongshan) were used to detect specific proteins according to the standard procedures. β-actin served as a loading control on the same membrane. Finally, the membranes developed with a Luminal Detection System (Santa Cruz).

### Senescence-associated β-galactosidase (SA-β-Gal) staining assay

Senescence Cell Histochemical Staining Kit (Sigma) was used to stain senescence cells both in vitro and in vivo. In vitro study, cultured cells were grown in 6-plates and transfected with DEC1 expression and control plasmids. After 72 h, the cells were stained in accordance with the manufacturer's procedure. In vivo study, the frozen sections were stained immediately after resection as described [57] and weakly counterstained with haematoxylin.

### Colony formation assay

EC9706 cells were co-transfected with pCMV6-XL5-DEC1 and pcDNA3.1 (−)/myc-His B, and cells that transfected with pcDNA3.1 (−)/myc-His B alone were used as control. The molar ratio of DEC1/pcDNA3.1/control was 10∶1∶1. Twenty-four hours after transfection, each kind of cells was seeded into six-well plate of 2000 cells/well and treated with 500 μg/mL G418 (Invitrogen). Two week later, cells was washed with PBS and fixed with formaldehyde. Then the cells were stained with Giemsa staining solution and clones were counted under an Olympus inverted microscope. The DEC1 expression was confirmed by western blot analysis.

### Cell growth assay

Twenty-four hours after transfection, cells were plated plated into 24-well plates at a density of 50 cells/mm^2^ in triplicate. Cells were harvested every day and cell numbers were counted with hematocytometer and Olympus inverted microscope.

### Statistical analysis

All statistical analysis was done using SPSS 18.0 for windows (SPSS Inc.). χ2 test was performed to compare of DEC1 expression among the consecutive stages of carcinogenesis and to assess the correlations between DEC1 expression in ESCC and clinicopathological characteristics. Survival analyses were carried out using Kaplan-Meier method with log-rank test. Values are expressed as mean ± s.d. A student two-tailed non-paired t-test was used to determine significant differences between treatment and control. P value <0.05 was considered statistically significant.

## Supporting Information

Figure S1 **Typical H&E staining of normal esophageal epithelia, ESCC and its precursor lesion.** (A) Normal esophageal epithelia. (B) Low grade intraepithelial neoplasia. (C) High grade intraepithelial neoplasia. (D) ESCC.
(TIF)

Table S1 **Summary of clinicopathological characteristics of 241 ESCC patients.**
(DOC)

## References

[1] Ferlay J, Shin HR, Bray F, Forman D, Mathers C (2010). Estimates of worldwide burden of cancer in 2008: GLOBOCAN 2008.. Int J Cancer.

[2] Jemal A, Bray F, Center MM, Ferlay J, Ward E, et al (2011). Global cancer statistics.. CA Cancer J Clin.

[3] Shirakawa Y, Naomoto Y, Kimura M, Kawashima R, Yamatsuji T (2000). Topological analysis of p21WAF1/CIP1 expression in esophageal squamous dysplasia.. Clin Cancer Res.

[4] Xue LY, Hu N, Song YM, Zou SM, Shou JZ (2006). Tissue microarray analysis reveals a tight correlation between protein expression pattern and progression of esophageal squamous cell carcinoma.. BMC Cancer.

[5] Wang GQ, Abnet CC, Shen Q, Lewin KJ, Sun XD (2005). Histological precursors of oesophageal squamous cell carcinoma: results from a 13 year prospective follow up study in a high risk population.. Gut.

[6] Cohen DJ, Ajani J (2011). An expert opinion on esophageal cancer therapy.. Expert Opin Pharmacother.

[7] Ohbu M, Kobayashi N, Okayasu I (2001). Expression of cell cycle regulatory proteins in the multistep process of oesophageal carcinogenesis: stepwise over-expression of cyclin E and p53, reduction of p21(WAF1/CIP1) and dysregulation of cyclin D1 and p27(KIP1).. Histopathology.

[8] Hiyoshi Y, Kamohara H, Karashima R, Sato N, Imamura Y (2009). MicroRNA-21 regulates the proliferation and invasion in esophageal squamous cell carcinoma.. Clin Cancer Res.

[9] Hayflick L, Moorhead PS (1961). The serial cultivation of human diploid cell strains.. Exp Cell Res.

[10] Hayflick L (1965). The Limited in Vitro Lifetime of Human Diploid Cell Strains.. Exp Cell Res.

[11] Harley CB, Futcher AB, Greider CW (1990). Telomeres shorten during ageing of human fibroblasts.. Nature.

[12] Ben-Porath I, Weinberg RA (2005). The signals and pathways activating cellular senescence.. Int J Biochem Cell Biol.

[13] Gewirtz DA, Holt SE, Elmore LW (2008). Accelerated senescence: an emerging role in tumor cell response to chemotherapy and radiation.. Biochem Pharmacol.

[14] Banito A, Rashid ST, Acosta JC, Li S, Pereira CF (2009). Senescence impairs successful reprogramming to pluripotent stem cells.. Genes Dev.

[15] Mooi WJ, Peeper DS (2006). Oncogene-induced cell senescence–halting on the road to cancer.. N Engl J Med.

[16] Kuilman T, Michaloglou C, Mooi WJ, Peeper DS (2010). The essence of senescence.. Genes Dev.

[17] Campisi J, d'Adda di Fagagna F (2007). Cellular senescence: when bad things happen to good cells.. Nat Rev Mol Cell Biol.

[18] Michaloglou C, Vredeveld LC, Soengas MS, Denoyelle C, Kuilman T (2005). BRAFE600-associated senescence-like cell cycle arrest of human naevi.. Nature.

[19] Braig M, Lee S, Loddenkemper C, Rudolph C, Peters AH (2005). Oncogene-induced senescence as an initial barrier in lymphoma development.. Nature.

[20] Chen Z, Trotman LC, Shaffer D, Lin HK, Dotan ZA (2005). Crucial role of p53-dependent cellular senescence in suppression of Pten-deficient tumorigenesis.. Nature.

[21] Collado M, Gil J, Efeyan A, Guerra C, Schuhmacher AJ (2005). Tumour biology: senescence in premalignant tumours.. Nature.

[22] Boudjelal M, Taneja R, Matsubara S, Bouillet P, Dolle P (1997). Overexpression of Stra13, a novel retinoic acid-inducible gene of the basic helix-loop-helix family, inhibits mesodermal and promotes neuronal differentiation of P19 cells.. Genes Dev.

[23] Shen M, Kawamoto T, Yan W, Nakamasu K, Tamagami M (1997). Molecular characterization of the novel basic helix-loop-helix protein DEC1 expressed in differentiated human embryo chondrocytes.. Biochem Biophys Res Commun.

[24] Guillaumond F, Lacoche S, Dulong S, Grechez-Cassiau A, Filipski E (2008). Altered Stra13 and Dec2 circadian gene expression in hypoxic cells.. Biochem Biophys Res Commun.

[25] Qian Y, Zhang J, Yan B, Chen X (2008). DEC1, a basic helix-loop-helix transcription factor and a novel target gene of the p53 family, mediates p53-dependent premature senescence.. J Biol Chem.

[26] Sun H, Taneja R (2000). Stra13 expression is associated with growth arrest and represses transcription through histone deacetylase (HDAC)-dependent and HDAC-independent mechanisms.. Proc Natl Acad Sci U S A.

[27] Collado M, Serrano M (2006). The power and the promise of oncogene-induced senescence markers.. Nat Rev Cancer.

[28] Turley H, Wykoff CC, Troup S, Watson PH, Gatter KC (2004). The hypoxia-regulated transcription factor DEC1 (Stra13, SHARP-2) and its expression in human tissues and tumours.. J Pathol.

[29] Chakrabarti J, Turley H, Campo L, Han C, Harris AL (2004). The transcription factor DEC1 (stra13, SHARP2) is associated with the hypoxic response and high tumour grade in human breast cancers.. Br J Cancer.

[30] Zheng Y, Jia Y, Wang Y, Wang M, Li B (2009). The hypoxia-regulated transcription factor DEC1 (Stra13, SHARP-2) and its expression in gastric cancer.. OMICS.

[31] Li Y, Zhang H, Xie M, Hu M, Ge S (2002). Abundant expression of Dec1/stra13/sharp2 in colon carcinoma: its antagonizing role in serum deprivation-induced apoptosis and selective inhibition of procaspase activation.. Biochem J.

[32] Giatromanolaki A, Koukourakis MI, Sivridis E, Turley H, Wykoff CC (2003). DEC1 (STRA13) protein expression relates to hypoxia- inducible factor 1-alpha and carbonic anhydrase-9 overexpression in non-small cell lung cancer.. J Pathol.

[33] Shi XH, Zheng Y, Sun Q, Cui J, Liu QH (2011). DEC1 nuclear expression: a marker of differentiation grade in hepatocellular carcinoma.. World J Gastroenterol.

[34] Wang W, Reiser-Erkan C, Michalski CW, Raggi MC, Quan L (2010). Hypoxia inducible BHLHB2 is a novel and independent prognostic marker in pancreatic ductal adenocarcinoma.. Biochem Biophys Res Commun.

[35] Chen X, Zhang W, Gao YF, Su XQ, Zhai ZH (2002). Senescence-like changes induced by expression of p21(waf1/Cip1) in NIH3T3 cell line.. Cell Res.

[36] Umar SB, Fleischer DE (2008). Esophageal cancer: epidemiology, pathogenesis and prevention.. Nat Clin Pract Gastroenterol Hepatol.

[37] Holmes RS, Vaughan TL (2007). Epidemiology and pathogenesis of esophageal cancer.. Semin Radiat Oncol.

[38] Chang MS, Lee HS, Lee BL, Kim YT, Lee JS (2005). Differential protein expression between esophageal squamous cell carcinoma and dysplasia, and prognostic significance of protein markers.. Pathol Res Pract.

[39] Kashyap MK, Marimuthu A, Kishore CJ, Peri S, Keerthikumar S (2009). Genomewide mRNA profiling of esophageal squamous cell carcinoma for identification of cancer biomarkers.. Cancer Biol Ther.

[40] Vallbohmer D, Lenz HJ (2006). Predictive and prognostic molecular markers in outcome of esophageal cancer.. Dis Esophagus.

[41] Braig M, Schmitt CA (2006). Oncogene-induced senescence: putting the brakes on tumor development.. Cancer Res.

[42] Gorgoulis VG, Halazonetis TD (2010). Oncogene-induced senescence: the bright and dark side of the response.. Curr Opin Cell Biol.

[43] Bartkova J, Rezaei N, Liontos M, Karakaidos P, Kletsas D (2006). Oncogene-induced senescence is part of the tumorigenesis barrier imposed by DNA damage checkpoints.. Nature.

[44] Prieur A, Peeper DS (2008). Cellular senescence in vivo: a barrier to tumorigenesis.. Curr Opin Cell Biol.

[45] Skinner HD, Sandulache VC, Ow TJ, Meyn RE, Yordy JS (2012). TP53 Disruptive Mutations Lead to Head and Neck Cancer Treatment Failure through Inhibition of Radiation-Induced Senescence.. Clin Cancer Res.

[46] Campo-Trapero J, Cano-Sanchez J, Palacios-Sanchez B, Llamas-Martinez S, Lo Muzio L (2008). Cellular senescence in oral cancer and precancer and treatment implications: a review.. Acta Oncol.

[47] Sarkisian CJ, Keister BA, Stairs DB, Boxer RB, Moody SE (2007). Dose-dependent oncogene-induced senescence in vivo and its evasion during mammary tumorigenesis.. Nat Cell Biol.

[48] Ogasawara S, Maesawa C, Tamura G, Satodate R (1995). Frequent microsatellite alterations on chromosome 3p in esophageal squamous cell carcinoma.. Cancer Res.

[49] Sakai N, Kajiyama Y, Iwanuma Y, Tomita N, Amano T (2010). Study of abnormal chromosome regions in esophageal squamous cell carcinoma by comparative genomic hybridization: relationship of lymph node metastasis and distant metastasis to selected abnormal regions.. Dis Esophagus.

[50] Tsai MH, Fang WH, Lin SH, Tzeng ST, Huang CS (2011). Mapping of genetic deletions on chromosome 3 in colorectal cancer: loss of 3p25-pter is associated with distant metastasis and poor survival.. Ann Surg Oncol.

[51] Campisi J (2005). Senescent cells, tumor suppression, and organismal aging: good citizens, bad neighbors.. Cell.

[52] Ohtani N, Takahashi A, Mann DJ, Hara E (2012). Cellular senescence: a double-edged sword in the fight against cancer.. Exp Dermatol.

[53] Decottignies A, d'Adda di Fagagna F (2011). Epigenetic alterations associated with cellular senescence: A barrier against tumorigenesis or a red carpet for cancer?. Semin Cancer Biol.

[54] Chandeck C, Mooi WJ (2010). Oncogene-induced cellular senescence.. Adv Anat Pathol.

[55] Kononen J, Bubendorf L, Kallioniemi A, Barlund M, Schraml P (1998). Tissue microarrays for high-throughput molecular profiling of tumor specimens.. Nat Med.

[56] Han Y, Wei F, Xu X, Cai Y, Chen B (2002). [Establishment and comparative genomic hybridization analysis of human esophageal carcinomas cell line EC9706].. Zhonghua Yi Xue Yi Chuan Xue Za Zhi.

[57] Debacq-Chainiaux F, Erusalimsky JD, Campisi J, Toussaint O (2009). Protocols to detect senescence-associated beta-galactosidase (SA-betagal) activity, a biomarker of senescent cells in culture and in vivo.. Nat Protoc.

