# Supplementary figures and images for: Overexpression of the DEC1 Protein Induces Senescence In Vitro and Is Related to Better Survival in Esophageal Squamous Cell Carcinoma
Source: PLoS One. 2012 Jul 23;7(7):e41862. doi: 10.1371/journal.pone.0041862 (PMC3402465; doi:10.1371/journal.pone.0041862)

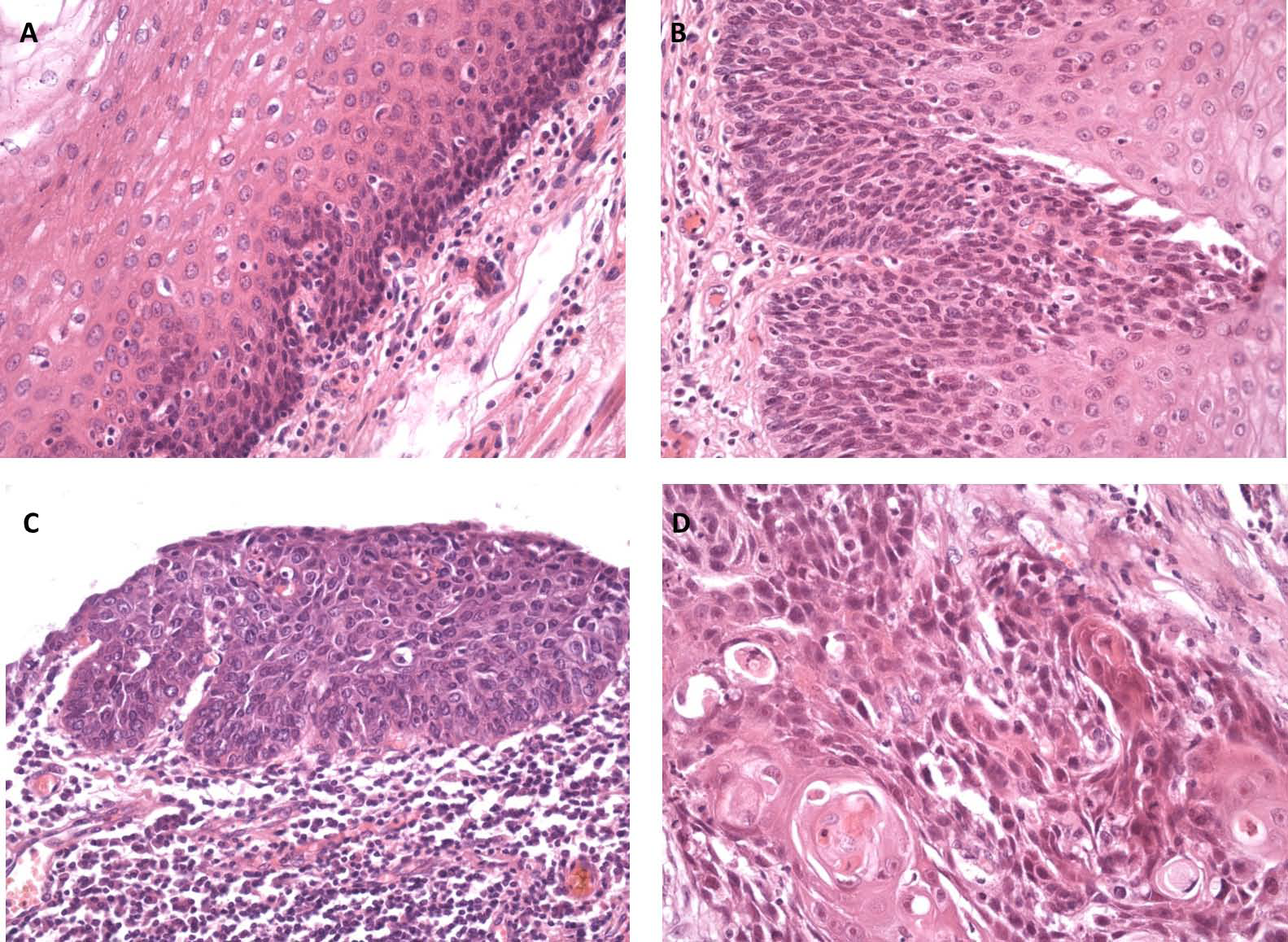

Supplement: Figure S1 — Typical H&E staining of normal esophageal epithelia, ESCC and its precursor lesion. (A) Normal esophageal epithelia. (B) Low grade intraepithelial neoplasia. (C) High grade intraepithelial neoplasia. (D) ESCC. (TIF) [file pone.0041862.s001.tif]
