# Supplementary material for: Overexpression of the DEC1 Protein Induces Senescence In Vitro and Is Related to Better Survival in Esophageal Squamous Cell Carcinoma
Source: PLoS One. 2012 Jul 23;7(7):e41862. doi: 10.1371/journal.pone.0041862 (PMC3402465; doi:10.1371/journal.pone.0041862)
Supplement: Table S1 — Summary of clinicopathological characteristics of 241 ESCC patients. (DOC) [file pone.0041862.s002.doc]

Table S1: Summary of clinicopathological characteristics of 241 ESCC patients.

|  | Low grade  intraepithelial  neoplasia | High grade  intraepithelial  neoplasia | ESCC |
| --- | --- | --- | --- |
| Cases | 162 (100%) | 162 (100%) | 241 (100%) |
| Age | | | |
| ＜60 | 82 (50.6%) | 96 (59.3%) | 130 (53.9%) |
| ≥60 | 80 (49.4%) | 66 (40.7%) | 111 (46.1%) |
| Gender | | | |
| Male | 119 (73.5%) | 118 (72.8%) | 177 (73.4%) |
| Female | 43 (26.5%) | 44 (27.2%) | 64 (26.6%) |
| Part of thoracic portion | | | |
| Upper | 23 (14.2%) | 20 (12.3%) | 35 (14.5%) |
| Middle | 103 (63.6%) | 110 (67.9%) | 154 (63.9%) |
| Lower | 36 (22.2%) | 32 (19.8%) | 52 (21.6%) |
| Differentiation | | | |
| Well | 41 (25.3%) | 25 (15.4%) | 48 (19.9%) |
| Moderate | 72 (44.4%) | 88 (54.3%) | 119 (49.4%) |
| Poor | 49 (30.2%) | 49 (30.2%) | 74 (30.7%) |
| Tumor embolus | | | |
| Negative | 137 (84.6%) | 143 (88.3%) | 205 (85.1%) |
| Positive | 25 (15.4%) | 19 (11.7%) | 36 (14.9%) |
| Depth of invasion | | | |
| T1 | 93 (57.4%) | 105 (64.8%) | 141 (58.5%) |
| T2 | 28(17.3%) | 21 (13.0%) | 35 (14.5%) |
| T3 | 41(25.3%) | 36 (22.2%) | 65 (27.0%) |
| Lymph metastasis | | | |
| Negative | 105 (64.8%) | 106 (65.4%) | 152 (63.1%) |
| Positive | 57 (35.2%) | 56 (34.6%) | 89 (36.9%) |
| pTNMs* | | | |
| Ⅰ | 72 (44.4%) | 81 (50.0%) | 106 (44.0%) |
| Ⅱa+Ⅱb | 66 (40.7%) | 56 (34.6%) | 95 (39.4%) |
| Ⅲ | 34 (14.8%) | 25 (15.4%) | 40 (16.6%) |

*pTNMs, pathological Tumor-Node-Metastasis. (AJCC, 6^th^ edition)
